# Supplementary material for: Vitamin D Status and Risk of All-Cause and Cause-Specific Mortality in Osteoarthritis Patients: Results from NHANES III and NHANES 2001–2018
Source: Nutrients. 2022 Nov 3;14(21):4629. doi: 10.3390/nu14214629 (PMC9655488; doi:10.3390/nu14214629)
Supplement: Supplementary file 1 [file nutrients-14-04629-s001.zip › nutrients-2009476-supplementary.pdf]

Table S1 HR (95% CIs) for all-cause and cause-specific mortality according to serum 25(OH)D concentrations among OA patients after excluding participants with history of cancers (n=3,640)

	Serum 25(OH)D concentrations (nmol/L)				Per one-unit increment in natural log-transformed 25(OH)D
	<25.0	25.0-49.9	50.0-74.9	≥75.0	
All-cause mortality					
Number of deaths/total	49/121	320/876	371/1,280	263/1,363	
Model 1 [a]	Ref.	0.41 (0.25, 0.68)	0.37 (0.22, 0.61)	0.31 (0.18, 0.54)	0.65 (0.50, 0.85)
Model 2 [b]	Ref.	0.44 (0.27, 0.71)	0.42 (0.26, 0.69)	0.38 (0.22, 0.65)	0.77 (0.59, 1.00)
Model 3 [c]	Ref.	0.47 (0.28, 0.77)	0.48 (0.30, 0.78)	0.43 (0.25, 0.74)	0.84 (0.64, 1.10)
CVD mortality					
Number of deaths	18	103	110	84	
Model 1 [a]	Ref.	0.27 (0.11, 0.62)	0.21 (0.09, 0.49)	0.19 (0.08, 0.46)	0.51 (0.29, 0.88)
Model 2 [b]	Ref.	0.27 (0.13, 0.58)	0.24 (0.11, 0.52)	0.24 (0.10, 0.56)	0.64 (0.37, 1.13)
Model 3 [c]	Ref.	0.27 (0.12, 0.61)	0.26 (0.12, 0.57)	0.25 (0.11, 0.55)	0.66 (0.40, 1.10)
Cancer mortality					
Number of deaths	7	46	70	56	
Model 1 [a]	Ref.	0.45 (0.17, 1.21)	0.50 (0.19, 1.28)	0.49 (0.18, 1.32)	0.86 (0.55, 1.36)
Model 2 [b]	Ref.	0.49 (0.18, 1.32)	0.62 (0.24, 1.61)	0.65 (0.24, 1.75)	1.03 (0.65, 1.63)
Model 3 [c]	Ref.	0.51 (0.18, 1.45)	0.65 (0.24, 1.78)	0.68 (0.24, 1.93)	1.04 (0.67, 1.64)
Other mortality					
Number of deaths	24	171	191	123	
Model 1 [a]	Ref.	0.56 (0.30, 1.04)	0.50 (0.28, 0.89)	0.38 (0.20, 0.72)	0.69 (0.50, 0.95)
Model 2 [b]	Ref.	0.57 (0.30, 1.08)	0.53 (0.29, 0.97)	0.44 (0.22, 0.85)	0.78 (0.56, 1.09)
Model 3 [c]	Ref.	0.70 (0.33, 1.45)	0.70 (0.35, 1.41)	0.58 (0.27, 1.26)	0.92 (0.64, 1.33)

NHANES, National Health and Nutrition Examination Survey; 25(OH)D, 25-Hydroxyvitamin D; OA, osteoarthritis; RA, rheumatoid arthritis; CVD, cardiovascular disease; HR, hazard ratio; CI, confidence interval; Ref., reference.

[a] Model 1 adjusted for age, gender, and race/ethnicity; [b] Model 2 further adjusted (from Model 1) for education, body mass index, family poverty income ratio, physical activity, smoking status, and drinking status; [c] Model 3 further adjusted (from Model 2) for diabetes mellitus, hypertension, cardiovascular disease, cancer, chronic lung disease, and renal disease

Table S2 HR (95% CIs) for all-cause and cause-specific mortality according to serum 25(OH)D concentrations among OA patients after excluding participants with history of CVD (n=3,636)

	Serum 25(OH)D concentrations (nmol/L)				Per one-unit increment in natural log-transformed 25(OH)D
	<25.0	25.0-49.9	50.0-74.9	≥75.0	
All-cause mortality					
Number of deaths/total	47/107	309/828	368/1,286	264/1,415	
Model 1 [a]	Ref.	0.30 (0.18, 0.51)	0.26 (0.15, 0.44)	0.25 (0.14, 0.46)	0.67 (0.51, 0.90)
Model 2 [b]	Ref.	0.33 (0.21, 0.52)	0.29 (0.18, 0.46)	0.30 (0.17, 0.53)	0.77 (0.58, 1.02)
Model 3 [c]	Ref.	0.35 (0.22, 0.56)	0.33 (0.21, 0.52)	0.34 (0.20, 0.58)	0.81 (0.62, 1.07)
CVD mortality					
Number of deaths	16	88	99	66	
Model 1 [a]	Ref.	0.17 (0.07, 0.42)	0.11 (0.05, 0.28)	0.12 (0.05, 0.33)	0.41 (0.23, 0.71)
Model 2 [b]	Ref.	0.17 (0.07, 0.41)	0.13 (0.06, 0.30)	0.17 (0.06, 0.45)	0.52 (0.28, 0.99)
Model 3 [c]	Ref.	0.18 (0.08, 0.42)	0.15 (0.07, 0.32)	0.18 (0.07, 0.44)	0.54 (0.29, 0.99)
Cancer mortality					
Number of deaths	8	56	78	68	
Model 1 [a]	Ref.	0.44 (0.18, 1.08)	0.41 (0.16, 1.02)	0.46 (0.18, 1.15)	0.82 (0.51, 1.31)
Model 2 [b]	Ref.	0.48 (0.20, 1.19)	0.47 (0.19, 1.15)	0.56 (0.23, 1.35)	0.94 (0.61, 1.44)
Model 3 [c]	Ref.	0.48 (0.18, 1.23)	0.47 (0.18, 1.19)	0.55 (0.21, 1.39)	0.91 (0.60, 1.39)
Other mortality					
Number of deaths	23	165	191	130	
Model 1 [a]	Ref.	0.42 (0.22, 0.80)	0.38 (0.21, 0.69)	0.34 (0.19, 0.61)	0.81 (0.60, 1.10)
Model 2 [b]	Ref.	0.44 (0.24, 0.81)	0.39 (0.23, 0.68)	0.38 (0.21, 0.67)	0.88 (0.66, 1.18)
Model 3 [c]	Ref.	0.52 (0.27, 0.98)	0.50 (0.28, 0.87)	0.47 (0.26, 0.85)	0.98 (0.74, 1.30)

NHANES, National Health and Nutrition Examination Survey; 25(OH)D, 25-Hydroxyvitamin D; OA, osteoarthritis; CVD, cardiovascular disease; HR, hazard ratio; CI, confidence interval; Ref., reference.

[a] Model 1 adjusted for age, gender, and race/ethnicity; [b] Model 2 further adjusted (from Model 1) for education, body mass index, family poverty income ratio, physical activity, smoking status, and drinking status; [c] Model 3 further adjusted (from Model 2) for diabetes mellitus, hypertension, cardiovascular disease, cancer, chronic lung disease, and renal disease.

Table S3 HR (95% CIs) for all-cause and cause-specific mortality according to serum 25(OH)D concentrations among OA patients after excluding participants who died within two years of follow-up (n=4,388)

	Serum 25(OH)D concentrations (nmol/L)				Per one-unit increment in
	<25.0	25.0-49.9	50.0-74.9	≥75.0	natural log-transformed 25(OH)D
All-cause mortality					
Number of deaths/total	48/126	370/995	453/1,522	335/1,745	
Model 1 [a]	Ref.	0.50 (0.32, 0.78)	0.43 (0.27, 0.67)	0.40 (0.25, 0.63)	0.73 (0.58, 0.91)
Model 2 [b]	Ref.	0.52 (0.33, 0.81)	0.47 (0.30, 0.73)	0.47 (0.29, 0.74)	0.84 (0.67, 1.05)
Model 3 [c]	Ref.	0.53 (0.34, 0.83)	0.51 (0.33, 0.78)	0.51 (0.33, 0.79)	0.90 (0.73, 1.11)
CVD mortality					
Number of deaths	16	119	142	93	
Model 1 [a]	Ref.	0.43 (0.19, 0.98)	0.32 (0.15, 0.71)	0.28 (0.12, 0.64)	0.55 (0.35, 0.86)
Model 2 [b]	Ref.	0.41 (0.18, 0.95)	0.36 (0.16, 0.79)	0.34 (0.15, 0.77)	0.71 (0.45, 0.12)
Model 3 [c]	Ref.	0.46 (0.20, 1.04)	0.41 (0.19, 0.92)	0.39 (0.18, 0.86)	0.75 (0.49, 1.15)
Cancer mortality					
Number of deaths	6	61	82	77	
Model 1 [a]	Ref.	0.68 (0.24, 1.91)	0.54 (0.19, 1.53)	0.72 (0.25, 2.03)	0.97 (0.60, 1.55)
Model 2 [b]	Ref.	0.73 (0.25, 2.07)	0.64 (0.22, 1.87)	0.88 (0.31, 2.51)	1.13 (0.73, 1.75)
Model 3 [c]	Ref.	0.67 (0.23, 1.98)	0.61 (0.20, 1.82)	0.84 (0.29, 2.46)	1.14 (0.74, 1.76)
Other mortality					
Number of deaths	26	190	229	165	
Model 1 [a]	Ref.	0.49 (0.28, 0.88)	0.46 (0.27, 0.78)	0.39 (0.22, 0.68)	0.76 (0.57, 1.01)
Model 2 [b]	Ref.	0.51 (0.29, 0.91)	0.48 (0.28, 0.80)	0.45 (0.25, 0.79)	0.85 (0.64, 1.15)
Model 3 [c]	Ref.	0.55 (0.31, 0.97)	0.54 (0.33, 0.90)	0.51 (0.29, 0.88)	0.94 (0.71, 1.26)

NHANES, National Health and Nutrition Examination Survey; 25(OH)D, 25-Hydroxyvitamin D; OA, osteoarthritis; RA, rheumatoid arthritis; CVD, cardiovascular disease; HR, hazard ratio; CI, confidence interval; Ref., reference.

[a] Model 1 adjusted for age, gender, and race/ethnicity; [b] Model 2 further adjusted (from Model 1) for education, body mass index, family poverty income ratio, physical activity, smoking status, and drinking status; [c] Model 3 further adjusted (from Model 2) for diabetes mellitus, hypertension, cardiovascular disease, cancer, chronic lung disease, and renal disease.

Table S4 HR (95% CIs) for all-cause and cause-specific mortality according to serum 25(OH)D concentrations among OA patients with further adjustment of fracture, lipid profiles, CRP, or lead

	Serum 25(OH)D concentrations (nmol/L)				Per one-unit increment in natural log-transformed 25(OH)D
	<25.0	25.0-49.9	50.0-74.9	≥75.0	
All-cause mortality					
Model 3 + Fracture [a]	Ref.	0.57 (0.36, 0.89)	0.54 (0.35, 0.85)	0.50 (0.31, 0.81)	0.82 (0.69, 0.99)
Model 3 + lipid profiles [b]	Ref.	0.49 (0.31, 0.76)	0.44 (0.29, 0.68)	0.43 (0.26, 0.70)	0.80 (0.65, 1.00)
Model 3 + CRP [c]	Ref.	0.44 (0.28, 0.68)	0.41 (0.26, 0.62)	0.39 (0.23, 0.66)	0.81 (0.63, 1.06)
Model 3 + lead [d]	Ref.	0.48 (0.31, 0.77)	0.44 (0.29, 0.69)	0.45 (0.27, 0.75)	0.82 (0.65, 1.05)
CVD mortality					
Model 3 + Fracture [a]	Ref.	0.55 (0.25, 1.21)	0.48 (0.23, 1.02)	0.42 (0.18, 0.99)	0.71 (0.47, 1.08)
Model 3 + lipid profiles [b]	Ref.	0.28 (0.13, 0.60)	0.25 (0.12, 0.52)	0.24 (0.11, 0.51)	0.62 (0.40, 0.98)
Model 3 + CRP [c]	Ref.	0.26 (0.12, 0.55)	0.21 (0.10, 0.43)	0.21 (0.09, 0.48)	0.48 (0.34, 0.98)
Model 3 + lead [d]	Ref.	0.31 (0.14, 0.69)	0.27 (0.12, 0.60)	0.28 (0.12, 0.63)	0.65 (0.42, 1.02)
Cancer mortality					
Model 3 + Fracture [a]	Ref.	0.75 (0.30, 1.90)	0.73 (0.29, 1.85)	0.77 (0.29, 2.00)	0.89 (0.64, 1.25)
Model 3 + lipid profiles [b]	Ref.	0.74 (0.29, 1.88)	0.67 (0.25, 1.75)	0.81 (0.32, 2.06)	1.01 (0.71, 1.45)
Model 3 + CRP [c]	Ref.	0.69 (0.26, 1.77)	0.64 (0.24, 1.73)	0.66 (0.25, 1.75)	0.89 (0.58, 1.36)
Model 3 + lead [d]	Ref.	0.70 (0.27, 1.76)	0.65 (0.25, 1.68)	0.76 (0.31, 1.90)	1.00 (0.69, 1.44)
Other mortality					
Model 3 + Fracture [a]	Ref.	0.50 (0.28, 0.89)	0.52 (0.31, 0.88)	0.49 (0.28, 0.85)	0.92 (0.74, 1.15)
Model 3 + lipid profiles [b]	Ref.	0.64 (0.36, 1.14)	0.60 (0.36, 1.00)	0.55 (0.31, 0.97)	0.88 (0.67, 1.14)
Model 3 + CRP [c]	Ref.	0.53 (0.30, 0.93)	0.54 (0.32, 0.89)	0.52 (0.29, 0.92)	0.97 (0.71, 1.32)
Model 3 + lead [d]	Ref.	0.60 (0.34, 1.04)	0.56 (0.34, 0.92)	0.56 (0.33, 0.95)	0.92 (0.70, 1.22)

NHANES, National Health and Nutrition Examination Survey; 25(OH)D, 25-Hydroxyvitamin D; OA, osteoarthritis; CVD, cardiovascular disease; CRP, C-reactive protein; HR, hazard ratio; CI, confidence interval; Ref., reference.

[a] Model further adjusted (from Model 3) for history of fracture, and 1090 subjects were lost due to missing related data; [b] Model further adjusted (from Model 3) for lipid profiles (i.e., the ratio of total cholesterol to HDL), and 45 subjects were lost due to missing related data; [c] Model further adjusted (from Model 3) for CRP, and 1,130 subjects were lost due to missing related data; [d] Model further adjusted (from Model 3) for lead, and 547 subjects were lost due to missing related data.
